# Effects of different removal methods of excess resin adhesive on the microleakage of alumina all-ceramic crowns

**DOI:** 10.1186/s12903-023-03581-z

**Published:** 2023-11-11

**Authors:** Haiyang Zhang, Hao Yu, Shangfei Jiang, Haidao Dong, Chengdong Yan, Hong Liu, Qing Li, Haiwei Jiang

**Affiliations:** 1 Department of Medicine, Jiangsu Vocational College of Medicine, NO.283 of Jiefang South Road, Yandu District, Yancheng, Jiangsu Province 224000 China; 2 Department of Stomatology, The First Hospital of Qiqihaer, No.700 South Pukui Road, Qiqihaer, Heilongjiang Province 161000 China

**Keywords:** Microleakage, All-ceramic crown, Resin cement, Excess removal, Marginal clearance

## Abstract

**Background:**

Microleakage is a common problem that affects the quality and longevity of all-ceramic crowns. It is influenced by factors such as the resin cement, crown margin design and curing technique. However, few studies focus on the effect of different methods of removing excess resin adhesive on the microleakage of all-ceramic crowns. This study aimed to compare two methods of removing excess resin adhesive (the small brush and sickle methods) on the microleakage of all-ceramic crowns with different marginal clearances.

**Methods:**

Forty extracted third molars were prepared with a 90° shoulder margin and randomly divided into four groups according to their marginal lift (30, 60, 90 or 0 μm). Procera alumina crowns were fabricated using computer-aided design/computer-aided modelling and cemented onto the teeth with 3 M RelyX Unicem (3 M Company, United States) resin cement. Excess resin cement was removed by either the small brush or the sickle scalpel method. The marginal adaptation was observed with a digital microscope. After thermal cycling of the teeth, microleakage was assessed using the dye penetration test under a stereomicroscope. The Mann–Whitney U test and Kruskal–Wallis H test were used to compare the microleakage scores among different groups.

**Results:**

The small-brush group showed significantly better marginal adaptation and lower microleakage scores than the sickle group (*p* < 0.05). There was no significant difference in the microleakage score (grade 0) among different marginal clearances within each group (*p* > 0.05).

**Conclusion:**

The small-brush method was more effective than the sickle scalpel method in reducing the microleakage of all-ceramic crowns with different marginal clearances. This method can improve the marginal adaptation and sealability of all-ceramic crowns, thus preventing secondary caries and other complications.

## Introduction

All-ceramic crowns are becoming more widely used in aesthetic restoration due to their excellent biocompatibility and low irritation to the gums [1]. Good marginal adaptation is essential for the quality and longevity of all-ceramic crowns [2]. In clinical practice, microleakage detection is often used to evaluate the marginal adaptability between materials and tooth structure [3]. Microleakage at the edge of the full crown is defined as the diffusion of bacteria, liquids, molecules, or ions between the full crown tooth and the adhesive bonding interface under the action of physical and chemical factors [4]. Microleakage can cause staining of the adhesive on the edge of the tooth, thus affecting aesthetics and accelerating the dissolution of the adhesive [5], particularly microleakage and the gap caused by partial loss or polymerisation of the material, which can easily form marginal caries between the material and the tooth [6]. So, the microleakage is an essential factor in the long-term failure of prostheses.

Microleakage is a common problem in dental restorative procedure [7]. There are many contributing factors for this phenomenon, including a mismatch thermal expansion of the bonding material and the tooth tissue, tooth tissue debris and residue not being cleaned, the aging of the adhesive and cold/hot expansion caused by the dynamic change in the oral cavity. The combination of these factors disrupts the contact between the tooth tissue and the adhesive, leading to gaps in the bonding site and promoting the formation of microleakage [8]. During the clinical crown bonding procedure, too much adhesive may be removed while eliminating excess bonding agent, resulting in a poor seal.

Previous studies have investigated the influence of various factors on the marginal fitness of all-ceramic crowns, such as the shape of the crown edge shoulder [9], the polymerisation angle [10] and the type of adhesive [11]. However, there is a lack of research on how different methods of removing excess resin cement affect microleakage at the crown edge.

In clinical practice, two common methods for removing excess resin cement are employed at the authors’ hospital (Peking University Stomatology Hospital, Beijing, China). One involves using a small brush to remove a large amount of excess resin cement after the full crown is bonded in place before using a sickle scaler to scrape a small amount of resin cement (the ‘small brush’ method). The other involves applying a curing light to a large amount of spilled excess cement for 2 s, scraping it with a sickle curette and then lighting it for a further 40 s (referred to as the ‘sickle’ method). The two methods differ greatly in operation sequence and the equipment used. However, there is no clear evidence on which approach is more effective or preferable for reducing microleakage.

Another factor that may influence the removal of excess resin cement is the marginal clearance of the crown, i.e. the vertical distance between the restoration’s internal surface and the preparation’s edge termination line. The marginal clearance of the full crown is an essential factor affecting the periodontal health of the abutment teeth [12]. Excessive marginal clearance may lead to increased plaque attachment, cement dissolution, microleakage and secondary caries and may even lead to tooth loss in the long term [13]. McLean et al. [14] proposed a marginal space (≤ 120 μm) to be clinically acceptable after a 5-year study of more than 1,000 restorations, which is also currently recognized by most clinicians as the standard. No study has reported whether removing excess adhesive, based on satisfying the marginal closeness of all-ceramic crowns, brings out more adhesive in restorations with large marginal gaps, thus exacerbating microleakage.

In this study, self-etching resin adhesive was chosen as the preferred conventional bonding system to explore the effect of different methods of removing excess adhesive on the microleakage of the full crown edge and to provide a standardized and feasible method for removing excess adhesive for the bonding of all-ceramic crowns with different edge gaps in clinical practice.

## Materials and methods

### Materials

A total of 40 third molars with complete crowns, no caries, no cracks and similar sizes was collected at the General Second Surgery Clinic of Peking University Stomatology Hospital (voluntarily donated by the patients after extraction). The calculus and periodontal tissue were carefully removed, and the teeth were stored in 1% chloramine solution ready to use at 4℃ for up to 3 months.

### Equipment

The equipment used included 3 M RelyX Unicem (3 M Company, United States); MANI dental needle (Japan); Silagum putty catalyst/Silais (DMG, Germany); Honigum-Light high-flow silicone rubber impression material (DMG, Germany); Die Stone Fujirock EP ultra anhydrite (GC, Tokyo, Japan); small brush (Densberg, Germany); water jet turbine and parallel grinder (bredent, Germany); Olympus SZ anatomy microscope and a MOTICAM1300 image acquisition instrument (Olympus, Japan); AutoCAD 2006 image processing software (Autodesk, USA); IBM SPSS Statistics 25.0 (IBM, USA); a laser confocal scanning electron microscope (Olympus, Japan); Procera computer-aided design/computer-aided manufacturing (CAD-CAM) scanner (Nobel Biocare, Switzerland); dental professional TC-801 cold and hot circulator (Shanghai Hualin Industrial Co., Ltd., Japan).

### Sample preparation and grouping

The sample size of this study was calculated based on the effect size and significance level of previous similar studies [15] using the G*Power software package version 3.1.9.2 (distributed by Heinrich Heine University, Düsseldorf, Germany). To ensure the validity and reliability of the study results, each group required at least five samples. Therefore, this study selected 40 third molars with complete crowns, no caries, cracks, and similar sizes. The teeth were randomly divided into two groups: the small brush group and the large sickle group, with 20 teeth in each group. Each group was then randomly divided into four subgroups (groups A, B, C and D) with five capsules in each group. A right-angle shoulder abutment tooth was prepared with a degree of polymerisation using a parallel tooth preparation instrument and a standard 6# carborundum turning needle. The occlusal surface was reduced by 2 mm with a 6° taper. An internal linear angle of 1.0 mm was also created. The same senior clinician performed the tooth preparation. After taking the impression, the super anhydrite was poured into the working model. The plaster model was scanned according to the operating instructions of the Procera CAD-CAM all-ceramic crown restoration system. After scanning the model, the edges of groups A, B and C restorationswere lifted at 30, 60 and 90 μm, respectively, and all-ceramic crown restorations were made (no edge lifting was applied to group D). Regarding the lifting method, termination points were made at points 3, 6, 9 and 12 of the crown, with no lifting performed at these specific points. Next, lifting was evenly conducted at all of the other positions. Four groups of all-ceramic crown samples with different edge gaps could be obtained, and the marginal gap intervals were measured for each set. All-ceramic crowns were sandblasted with aluminium oxide and steam cleaned. The prepared tooth were immersed in a 0.1% thymol solution, cleaned and dried and prepared for use. The images were observed and captured using an anatomical microscope and a MOTICAM1300 image acquisition instrument. The specimen table was rotated and the edge clearance value was recorded every 200 μm along the crown and neck edge by the buccal side, lingual side, mesial-middle and distal-middle, and the average value and standard deviation were calculated (for groups A, B and C, only the area with the edge raised for measurement was selected). A minimum of 40 points is guaranteed for each group.

The method for removing excess resin adhesive in the small brush group was as follows. Once the full crown was bonded in place, a small brush was used to remove a large amount of excess resin adhesive, followed by 40 s of irradiation, and the sickle curette was used to scrape a small amount of resin adhesive.

The method of removing excess resin adhesive from the sickle group was as follows. For a large amount of spilled excess adhesive, it was illuminated for 2 s, scraped with a sickle curette and then lit for a further 40 s. Finally, the changes in edge adaptability of the all-ceramic crowns after bonding using the same method were measured and recorded.

### Simulated aging and microleakage assessment of the thermal cycling process

In an oral environment, changes in oral humidity and temperature affect the performance of the bonding material and may lead to expansion and aging of the adhesive; this may disrupt the bond between the tooth tissue and the adhesive and promote microleakage. The thermal cycling process is often used to simulate changes in oral temperature [16]. Therefore, after cementing, all the models were placed in a hot/cold circulation machine and then exposed to a cold-water bath at 5℃ for 60 s, followed by a 15 s immersion in warm-water bath at room temperature. Finally, the models were exposed to a hot water bath at 55℃ for 60 s. A total of 3,500 such cycles were conducted. At the end of the thermal cycling test, each sample was washed and dried, and two layers of oily nail polish were evenly coated on the tooth tissue area 1 mm away from the edge, dried thoroughly and soaked in 2% methylene dye for 24 h at an ambient temperature of 37℃. The excess dye was rinsed with flowing water to remove nail polish.

The all-ceramic crown specimens were cut continuously along the buccal and lingual diameter with a cutting machine; the thickness of each section was 1 mm. Two sections were selected from one specimen and observed under a 40x-magnification microscope. Two observation points were chosen for each section, i.e. four observation points for each group, to evaluate the level of microleakage.

### Main observations

The main index served as the grade of edge microleakage; the grade evaluation was divided into five grades [17]: grade 0 – without microleakage; grade 1 – microleakage was within one-third of the length of the shoulder; grade 2 – microleakage was more than one-third but less than two-thirds of the shoulder; grade 3 – dye penetration exceeded two-thirds, but did not reach the shaft wall; grade 4 – the dye infiltrated into the axial pulp wall at the bottom of the hole.

### Statistical analysis

The statistical processing was performed using IBM SPSS Statistics 25.0 software. The edge clearance values were expressed as mean ± standard deviation, and the data were analysed using the one-way analysis of variance method. The microleakage scores were expressed as median (interquartile range), and the data were analysed using the Kruskal–Wallis H test or Mann–Whitney U test, depending on the number of groups compared. The level of significance was set at *p* < 0.05.

## Results

### Edge adaptability of standard model all-ceramic crowns before bonding

After creating the all-ceramic crown restoration model, the edge measurements were conducted. Before bonding, each group met the minimum standard of edge suitability of < 200 μm, which was slightly higher than the clinically acceptable standard of 120 μm proposed by McLean et al. [14]. This criterion was chosen because it was more feasible and realistic for the CAD-CAM system and clinical practice adopted by the authors, which was intentionally selected to help avoid seating issues during the bonding process, especially due to the hydrostatic pressure of the luting agent, which could prevent the full seating of the crown if the adaptation was excessively tight. There was no statistically significant difference between the small brush group and the large sickle group in terms of marginal adaptation at different marginal gaps, indicating that the two groups were comparable (Table 1).

**Table 1:** Adaptability of the edges of all ceramic crowns before bonding (± s, n = 5, µm)

	Edge gap (µm)	Buccal side			Lingual side			Mesial middle			Distal middle		
		Small brush group	Large sickle group	P Value	Small brush group	Large sickle group	P Value	Small brush group	Large sickle group	P Value	Small brush group	Large sickle group	P Value
A group (n = 5)	30	81.5 ± 7.4	83.0 ± 10.8	0.474	81.9 ± 8.3	82.2 ± 9.8	0.892	87.3 ± 5.8	86.7 ± 9.6	0.751	82.0 ± 6.4	84.9 ± 7.1	0.056
B group (n = 5)	60	97.1 ± 11.5	100.2 ± 8.6	0.057	112.3 ± 0.9	110.3 ± 4.3	0.137	107.2 ± 7.3	109.2 ± 7.8	0.670	95.8 ± 9.4	96.4 ± 10.5	0.961
C group (n = 5)	90	137.1 ± 11.6	137.3 ± 12.9	0.940	136.8 ± 6.7	136.8 ± 12.9	0.984	140.4 ± 10.3	138.1 ± 9.9	0.312	142.5 ± 9.90	141.2 ± 10.3	0.564
D group (n = 5)	0	58.9 ± 10.7	59.9 ± 9.6	0.665	58.8 ± 8.2	60.3 ± 7.8	0.160	55.0 ± 7.5	57.5 ± 7.8	0.534	62.6 ± 8.5	60.8 ± 9.1	0.384

### Adaptability of the all-ceramic crown edge after bonding

The samples with different edge gaps were treated using the same adhesive and different methods of removing excess adhesive, and the changes in edge adaptability after bonding were measured. The results are shown in Table 2. The difference in marginal adaptation in the small brush group was statistically significant (p < 0.05) in the buccal, lingual and distal and mesial four sites compared to the large sickle group. (*p* < 0.05).

**Table 2:** Changes in edge adaptability of all ceramic crowns after bonding (± s, µm)

	Edge gap (µm)	Buccal side			Lingual side			Mesial middle			Distal middle		
		Small brush	Large sickle group	P Value	Small brush	Large sickle group	P Value	Small brush	Large sickle group	P Value	Small brush	Large sickle group	P Value
A group (n = 5)	30	92.8 ± 11.5	98.8 ± 8.6	0.01	92.7 ± 9.7	98.5 ± 8.4	0.005	95.8 ± 10.2	104.5 ± 8.0	< 0.001	87.5 ± 2.4	97.2 ± 5.5	< 0.001
B group (n = 5)	60	108.7 ± 8.9	116.8 ± 9.6	< 0.001	118.2 ± 7.6	127.2 ± 6.6	< 0.001	125.1 ± 9.1	135.1 ± 9.7	< 0.001	115.1 ± 9.7	127.6 ± 8.6	< 0.001
C group (n = 5)	90	160.6 ± 11.6	170.3 ± 11.3	< 0.001	154.8 ± 7.7	165.3 ± 10.2	< 0.001	150.5 ± 7.6	162.8 ± 11.7	< 0.001	160.6 ± 10.8	169.6 ± 8.4	< 0.001
D group (n = 5)	0	60.8 ± 10.5	70.3 ± 8.1	< 0.001	662 ± 10.3	74.0 ± 6.7	< 0.001	63.7 ± 9.2	70.2 ± 7.8	0.001	70.3 ± 6.0	74.7 ± 5.4	0.001

### Effects of different operations on microleakage at the crown edge

At the end of the thermal cycling, the penetration depth of marginal dye was compared between the groups, and the microleakage grade of crown edges was calculated for both groups. The results are shown in Table 3. The incidence of no microleakage (grade 0) in the small brush group was 30%, significantly higher than that in the sickle group (5%). There was a statistically significant difference in microleakage grade between the small brush group and the large sickle group (*p* < 0.05).

**Table 3:** Comparison of penetration depth of each group of edge dyes after cold and hot cycles (pcs.)

group	0 level	1level	2level	3level	4 level	P value
small brush group(n = 20)	6	9	3	2	0	0.029
large sickle group(n = 20)	1	5	8	6	0	
Differences between groups P-value	0.037					

### Effects of two operations on the marginal microleakage of crowns with different marginal gaps

To evaluate the impact of the two operations on microleakage at the crown edge of different edge gaps, the samples without microleakage (grade 0) in the two sample groups under different edge gaps were counted, and the results are shown in Table 4. Using the same cleaning method, no statistical significance (*p* > 0.05) was observed in the difference for microleakage between different edge clearance values, which implied that the edge clearance had no significant impact on the operation effect of clearing excess adhesive.

**Table 4:** Statistics of samples without micro leakage (level 0) under different edge gap conditions in each group (pcs.)

group	Edge gap (µm)	small brush group(n = 20)	large sickle group(n = 20)
A group(n = 5)	30	2*	0*
B group(n = 5)	60	1*	0*
C group(n = 5)	90	1*	0*
D group(n = 5)	0	2	1

Note: *: compared to group D, p > 0.05

## Discussion

The main finding of this study was that the small brush method was more effective than the sickle method in reducing microleakage at the crown edge, regardless of the marginal clearance of the crown. This finding suggests that using a small brush to remove excess resin cement can improve the all-ceramic crowns’ marginal fitness and sealability and thus may prevent secondary caries and other complications.

One possible explanation for this results is that the use of a small brush can remove excess resin cement more gently and thoroughly than using a sickle curette, which may cause damage to the crown or tooth structure or leave some residual cement. Moreover, using a small brush can avoid premature light curing of excess resin cement, which may interfere with the adaptation of the crown and create gaps at the margin [18]. These factors may affect the integrity and durability of the edge seal, which are essential for maintaining pulp health and prolonging the life of the prosthesis [19].

Another finding of this study was that there was no significant difference in microleakage among different marginal clearances as long as they were within the experimental criterion of < 200 μm. This finding indicates that marginal clearance is not a decisive factor for microleakage, provided it is within a clinically acceptable range. However, this finding contradicts previous studies that reported a positive correlation between marginal clearance and microleakage [19, 20]. One possible reason for this discrepancy is that the experimental criterion of < 200 μm was slightly higher than the literature standard of 120 μm [14], which may have reduced the sensitivity of detecting differences in microleakage across marginal clearances. Therefore, further studies are needed to confirm whether marginal clearance impacts microleakage at lower levels.

The authors acknowledge that this study has some limitations that may affect its generalisability and validity. First, the sample size was relatively small (*n* = 20 per group), which may limit the statistical power and representation of the results. Second, the experimental criterion of < 200 μm was based on the authors’ experimental design and not on the literature standard of 120 μm [14], which may raise some questions about its clinical relevance and applicability. Third, this study only used one type of resin cement (3 M RelyX Unicem) and one type of all-ceramic crown (Procera CAD-CAM), which may not reflect the performance of other types of materials and techniques in clinical practice. Fourth, this study only used one method of measuring microleakage (the dye penetration method), which may have some drawbacks, such as poor quantification, sample destruction and measurement accuracy [21]. Therefore, future studies should consider increasing the sample size, using different types of materials and techniques and comparing different methods of measuring microleakage.

The main contribution of this study is that it provides experimental evidence for the effect of different methods of removing excess resin cement on the microleakage of all-ceramic crowns with different marginal clearances. This evidence can help clinicians choose the best method for removing excess resin cement and improve the quality and longevity of their restorations. This study also adds to the existing literature on the factors that influence microleakage and provides some directions for future research.

## Conclusion

Large amounts of excess adhesive should be removed with a small brush, followed by illumination treatment for 40 s using an ultraviolet curing lamp. After the surface adhesive has hardened, a sickle curette should be used to remove the excess adhesive, which can effectively reduce the occurrence of microleakage at the crown edge. This method is best suited for clinical operations.

## Data Availability

The datasets used and/or analyzed during the current study are available from the corresponding author upon reasonable request.
